# Are mGluR2/3 Inhibitors Potential Compounds for Novel Antidepressants?

**DOI:** 10.1007/s10571-022-01310-8

**Published:** 2022-11-29

**Authors:** Yajie Jiang, Manshu Zou, Tingting Ren, Yuhong Wang

**Affiliations:** 1 Institute of Innovation and Applied Research in Chinese Medicine, Hunan University of Chinese Medicine, Changsha, China; 2 Hunan Key Laboratory of Traditional Chinese Medicine Prevention & Treatment of Depressive Diseases, Hunan University of Chinese Medicine, Changsha, China

**Keywords:** mGluR2/3, Depression, Antagonists, Negative allosteric modulators, Antidepressants

## Abstract

Depression is the most common mental illness characterized by anhedonia, avolition and loss of appetite and motivation. The majority of conventional antidepressants are monoaminergic system selective inhibitors, yet the efficacies are not sufficient. Up to 30% of depressed patients are resistant to treatment with available antidepressants, underscoring the urgent need for development of novel therapeutics to meet clinical needs. Recent years, compounds acting on the glutamate system have attracted wide attention because of their strong, rapid and sustained antidepressant effects. Among them, selective inhibitors of metabotropic glutamate receptors 2 and 3 (mGluR2/3) have shown robust antidepressant benefits with fewer side-effects in both preclinical and clinical studies. Thus, we here attempt to summarize the antidepressant effects and underlying mechanisms of these inhibitors revealed in recent years as well as analyze the potential value of mGluR2/3 selective inhibitors in the treatment of depression.

## Introduction

According to estimates from the World Health Organization, over 300 million people are diagnosed with depression globally, with a prevalence of 4.4% (WHO 2017). As a chronic, recurring mental disorder, depression is featured by clinical symptoms such as continuous black mood, anhedonia, avolition and changes in appetite, causing enormous disability (Organization 2016; Zapata and Pearlstein 2022). The prescribed medications currently are mainly first-generation (monoamine oxidase inhibitors [MAOIs] and tricyclic antidepressants [TCAs]) and second-generation antidepressants (selective serotonin reuptake inhibitors [SSRIs] and serotonin-norepinephrine reuptake inhibitors [SNRIs]). Among them, SSRIs are usually used as the “first-line” antidepressants for depression treatment, owing to their high safety and fewer side-effects (Koenig and Thase 2009; Trivedi et al. 2006). However, these available antidepressants have several undesirable limitations. Firstly, more than 30% of the depressed patients do not respond to these drugs, thereby being referred to as "treatment-resistant depression (TRD)". Secondly, it takes weeks or even months to reach full effectiveness after the first dose of these drugs. Thirdly, some symptoms of depression are difficult to be treated effectively with available antidepressants (Fabbri et al. 2021; Nutt et al. 2007). All of these limitations highlight the critical need to develop more effective and rapid-acting interventions to alleviate all symptoms of depression

Accumulating evidence from postmortem and imaging studies suggests that glutamate system dysregulation plays a vital role in mood disorders including depression (Sanacora et al. 2004; Yildiz-Yesiloglu and Ankerst 2006). Changes in glutamate levels in different clinical samples from depressed individuals have been confirmed (Levine et al. 2000; Mitani et al. 2006), along with the specific proteins related to synaptic connections and glutamate receptors (Duric et al. 2013). Additionally, evidence also shows that targeting glutamate system has robust and rapid-acting antidepressant effects. Therefore, glutamate system has been widely concerned as the potential target for the treatment of depression as well as TRD (Pilc et al. 2013; Sanacora et al. 2008). Clinical investigations have revealed that ketamine, a glutamate system modulator, has robust anti-depressive effects, though not all depressed patients respond to this compound (Berman et al. 2000; Murrough et al. 2013). Ketamine also has serious adverse effects, including psychotomimetic effects and abuse liability (Sos et al. 2013), which restrict its clinical utility. However, the robust antidepressant effects of ketamine have altered our expectations regarding the speed of antidepressant response and bolstered efforts to identify more rapid-acting treatments.

Glutamate is one of the classical excitatory neurotransmitters in the central nervous system. To our knowledge, it mainly exerts its effects through two principal classes of the receptor, called ionotropic glutamate receptors (iGluRs) and metabotropic glutamate receptors (mGluRs), respectively (Nakanishi 1992). Based on the affinity for glutamate and its analogs, iGluRs are divided into three subtypes: α-amino-3-hydroxy-5-methyl-isoxazole-4-proprionic acid receptor (AMPAR), N-methyl-D-aspartate receptor (NMDAR) and kainate receptor. These ion channel complexes are responsible for mediating fast cation flux and synaptic transmission across the postsynaptic neuronal membrane. mGluRs have eight members (mGluR1-8) (Nakanishi 1994; Nicoletti et al. 2011) and based on the sequence homology, G-protein coupling, ligand selectivity and function of mGluRs, they are sub-classified into three groups: group I (mGluR1 and mGluR5), group II (mGluR2 and mGluR3) and group III (mGluR4 and mGluR6-8) (Niswender and Conn 2010). Extensive studies have shown that mGluRs are implicated in the pathology of depression. Compounds acting on mGluRs are considered as potential agents for depression treatment. Notably, both preclinical and clinical studies have demonstrated that agents targeting mGluR2/3, including the antagonist and negative allosteric modulator (NAM), possess fast and sustained antidepressant-like effects similar to that of ketamine though part of these compounds are not launched into clinical trials yet. Furthermore, these agents are even efficacious for TRD in animal models. In this brief review, we aim to summarize and update published preclinical and clinical studies investigating the antidepressant effects of mGluR2/3 selective inhibitors and analyze the potential value of these compounds as novel antidepressants.

### mGluR2/3 Signaling in the Pathogenesis of Depression

As the receptor of the neurotransmitter glutamate, mGluR2/3 is distributed in brain regions which are linked to social behavior and emotion regulation, such as the prefrontal cortex (PFC), anterior cingulate cortex, thalamus, amygdala and hippocampus (Matosin et al. 2014; Wright et al. 2001), indicating a modulatory role in depression. While group II mGluRs are predominantly located presynaptically where they function as auto- and hetero-receptors and inhibit the release of glutamate and other neurotransmitters, mGluR3 is also found in postsynaptic and glial localizations (Petralia et al. 1996; Tamaru et al. 2001). mGluR2/3 belongs to class C G-protein coupled receptor which couples to Gi/o proteins and then inhibit adenylyl cyclase and directly regulate ion channels and other downstream signaling molecules via the release of G_βγ_ subunits. Additionally, mGluR2/3 also activates other signaling pathways, including MAPK and phosphatidylinositol 3-kinase (PI3 kinase) pathways (Iacovelli et al. 2002), resulting in changes of the expression of downstream genes such as BDNF, PSD95 and Synapsin I. Activation of mGluR2/3 directly affects glutamate levels and synaptic plasticity (Machado-Vieira et al. 2009). These validated effects of mGluR2/3 in modulating glutamatergic signaling make them potential targets for developing novel pharmacotherapies for depression treatment.

Several brain regions, including PFC, striatum, nucleus accumbens (NAc), thalamus, hippocampus and amygdala, were proven to be involved in regulation of the mood, cognition and depression behavior (Nicoletti et al. 2011; Wright et al. 2001). Coincidentally, studies have revealed that the expression of mGluR2/3 is altered in these regions in both depressed patients and animal models (Feyissa et al. 2010; Pytka et al. 2016; Wang et al. 2015). For instance, mGluR2/3 was increased in PFC and hippocampus in the mice reared under isolated conditions (Kawasaki et al. 2011) and in the postmortem PFC of depressed patients (Feyissa et al. 2010), suggesting that elevated function of mGluR2/3 might be the etiological hallmark of depression.

### The Antidepressant Effects of mGluR2/3 Antagonists

It has been confirmed that mGluR2/3 antagonists increase synaptic glutamate levels, commensurately boosting AMPA receptor transmission and firing rates and extracellular monoamine levels. Multiple mGluR2/3 antagonists have been studied, as listed in Table 1, and they have all demonstrated beneficial effects on depression. mGluR2/3 antagonists, including MGS0039, LY341495 and LY3030371,display fast and sustained antidepressant-like responses in depression models (Campo et al. 2011; Chaki et al. 2004; Dwyer et al. 2013; Fukumoto et al. 2016; Joffe et al. 2020; Koike et al. 2013b; Podkowa et al. 2015), compared to the conventional antidepressants which have a substantial delay in the therapeutic onset. For instance, MGS0039 and LY341495 have been shown to manifest antidepressant-like effects as early as one day after administration, without any therapeutic delay (Dong et al. 2017; Dwyer et al. 2013). What’s more, the antidepressant-like effects of a single injection of mGluR2/3 antagonists last for at least a week, illustrating the prolonged effects of these antagonists (Dong et al. 2017; Dwyer et al. 2013). Interestingly, the sustained antidepressant effects might be attributed to persistent recovery in synaptic plasticity, rather than pharmacokinetic profile of the antagonists, as they are quickly cleared from the body within one day (Nakazato 2009; Ornstein et al. 1998).

**Table 1:** Summary of the preclinical effects, effective doses of mGluR2/3 inhibitors in animals

Compounds		Doses/ Administration	Animals	Effects in the experimental models	References
mGluR2/3 antagonists	MGS0039	0.3–3 mg/kg, i.p	Rat (Sprague–Dawley)	Show anti-antidepressant-like in FST and TST	Chaki et al. (2004)
		1 mL/kg, i.p	Mouse (NIH-Swiss)	Show anti-antidepressant-like in FST	Gleason et al. (2013)
		1 mg/kg, i.p	Mouse (ICR)	Show anti-antidepressant-like in TST	Koike et al. (2011b)
		1 and 3 mg/ kg, i.p	Mouse (C57BL/6 J)	Show anti-antidepressant-like in TST	Pałucha-Poniewiera et al. (2010)
		10 mg/kg, i.p	Rat (Sprague–Dawley)	Show anti-antidepressant-like in TST of learned helplessness model	Yoshimizu et al. (2006)
		1 and 3 mg/kg, i.p	Rat (Sprague–Dawley)	Show anti-antidepressant-like in open field test in olfactory bulbectomy model of depression	Pałucha-Poniewiera et al. (2010)
		1 mg/kg, i.p	Mouse (ddy)	Exert the anti-antidepressant-like by blocking dopamine release in prefrontal of chronic corticosterone-treated mice	Ago et al. (2013)
		1 mg/kg, i.p	Mouse (ddy)	Decrease the immobility time of isolation-reared mice in FST	Kawasaki et al. (2011)
		1 mg/kg, i.p	Mouse (C57BL/6 J)	Exert rapid and sustained antidepressant-likes in the social defeat stress model through BDNF-TrkB signaling	Dong et al. (2017)
	LY341495	0.1–3 mg/kg, i.p	Rat (Sprague–Dawley)	Have dose-dependent antidepressant-like effect in FST	Chaki et al. (2004)
		5 mL/kg, i.p	C57BL/6 J	Show dose-dependently reduced immobility time in FST	Campo et al. (2011)
		1 mg/kg, i.p	Mouse (NIH-Swiss)	Have dose-dependent antidepressant-like effect in FST	Gleason et al. (2013)
		0.3–3 mg/kg, i.p	Mouse (NMRI)	Have dose-dependent antidepressant-like effect in FST	Bespalov et al. (2008)
		1 mg/kg, i.p	Mouse (ICR)	Show antidepressant-like effect in TST and novelty-suppressed feeding test	Koike et al. (2013a)
		3 mg/kg, i.p	Mouse (CD-1)	Show antidepressant-like effect in TST through increasing the number of active dopamine neurons in the ventral tegmental area, increasing extracellular levels of dopamine in the nucleus accumbens and prefrontal cortex, and enhancing the locomotor stimulatory effects of dopamine D2/3 receptor agonist quinpirole	Witkin et al. (2016)
		5 mL/kg, i.p	Mouse (helpless)	Exert antidepressant-like effect in TST	Campo et al. (2011)
		0.3–3 mg/kg, i.p	RAT (Wistar)	Reduce immobility in the mouse FST	Bespalov et al. (2008)
		0.3 mg/kg, i.p	Mouse (ddy)	Exert the anti-antidepressant-like by blocking dopamine release in the prefrontal of chronic corticosterone-treated mice	Ago et al. (2013)
		0.3–3 mg/kg, i.p	Rat (Sprague–Dawley)	Reduce immobility in the mouse FST	Koike et al. (2013b)
		1–3 mg/kg, i.p	Rat (Sprague–Dawley)	Reduce immobility in the mouse FST	Iijima et al. (2013)
		3 mg/kg, i.p	Rat (Sprague–Dawley)	Produce rapid and robust antidepressant-like in sucrose preference	Dwyer et al. (2013)
	LY3030371	1 or 2 mL/kg, i.p	Rat (Sprague–Dawley)	Show antidepressant-like effect in sucrose preference	Witkin et al. (2017a)
		1–10 mg/kg, i.p	Mouse (NIH-Swiss)	Show antidepressant-like effect in FST	Chappell et al. (2016)
	RO1, RO2	10 mL/kg, i.p	Mouse (NIH-Swiss)	Show antidepressant-like effect in FST	Gleason et al. (2013)
mGluR2/3 NAM	RO4491533	1,3,10,30,100 mg/kg, p.o	C57BL/6 J	Reduce immobility time in FST	Campo et al. (2011)
			Mouse (helpless)	Show antidepressant-like effect in TST	Campo et al. (2011)
	RO4432717	10 mg/kg, p.o	Rat (Sprague–Dawley)	Increase long-term potentiation in dentate gyrus and improve cognitive, learning behaviors in rat	Goeldner et al. (2013)
mGluR2 NAM	VU6001966	10 mg/kg, i.p	Mouse	Increase latency to immobility and decrease total immobile time in FST; reverse anhedonia induced by chronic corticosterone treatment or exposure to chronic variable stress	Joffe et al. (2020)
mGluR3 NAM	VU650786	10 mg/kg,i.p	Rat (Sprague–Dawley)	Inhibit marble burying in mice, decrease immobility in FST	Engers et al. (2015)
		10 μL/g,i.p	Mouse (C57BL/6 J)	Prevent motivational deficits induced by acute stress, increase latency to immobility and decrease total immobile time in FST and TST; reverse anhedonia induced by chronic CORT treatment or exposure to CVS	Joffe et al. (2019)
	VU6010572	3 mg/kg, i.p	Mouse (CD-1)	Reduce immobility time in TST	Engers et al. (2017)

As discussed above, one-third of depressed patients show resistance to the treatment of conventional antidepressants. These conventional drugs show limited efficacy in rodent models of conventional antidepressant-resistant such as learned helplessness (Yoshimizu et al. 2006) and corticosterone-treated animal models (Ago et al. 2013; Iijima et al. 2010). However, some mGluR2/3 antagonists, such as LY341495, potentiate stress resilience in rodents (Highland et al. 2019) and induce antidepressant effects in the SSRI-resistant CD-1 mice (Witkin et al. 2016). LY341495 was also shown to increase glutamate outflow in the limbic regions and PFC (Hascup et al. 2010; Xi et al. 2002), increase mTOR pathway signaling and thereby promote the expression of the synaptic proteins GluR1, PSD-95 and Synapsin I (Dwyer et al. 2012; Koike et al. 2011a). These preclinical findings indicate that mGluR2/3 antagonists may be effective for TRD which currently prescribed antidepressants are not. Furthermore, LY3020371 shows a ketamine-like antidepressant effect in the forced swimming test (FST) (Witkin et al. 2017a), but doesn't produce any ketamine-like adverse effects (Witkin et al. 2017b). All these studies demonstrate that mGluR2/3 antagonists possess prolonged, fast-acting antidepressants with relatively high safety, indicating a promising value in the treatment of depression.

### The Antidepressant-Like Effects of mGluR2/3 NAMs

**Fig. 1:**
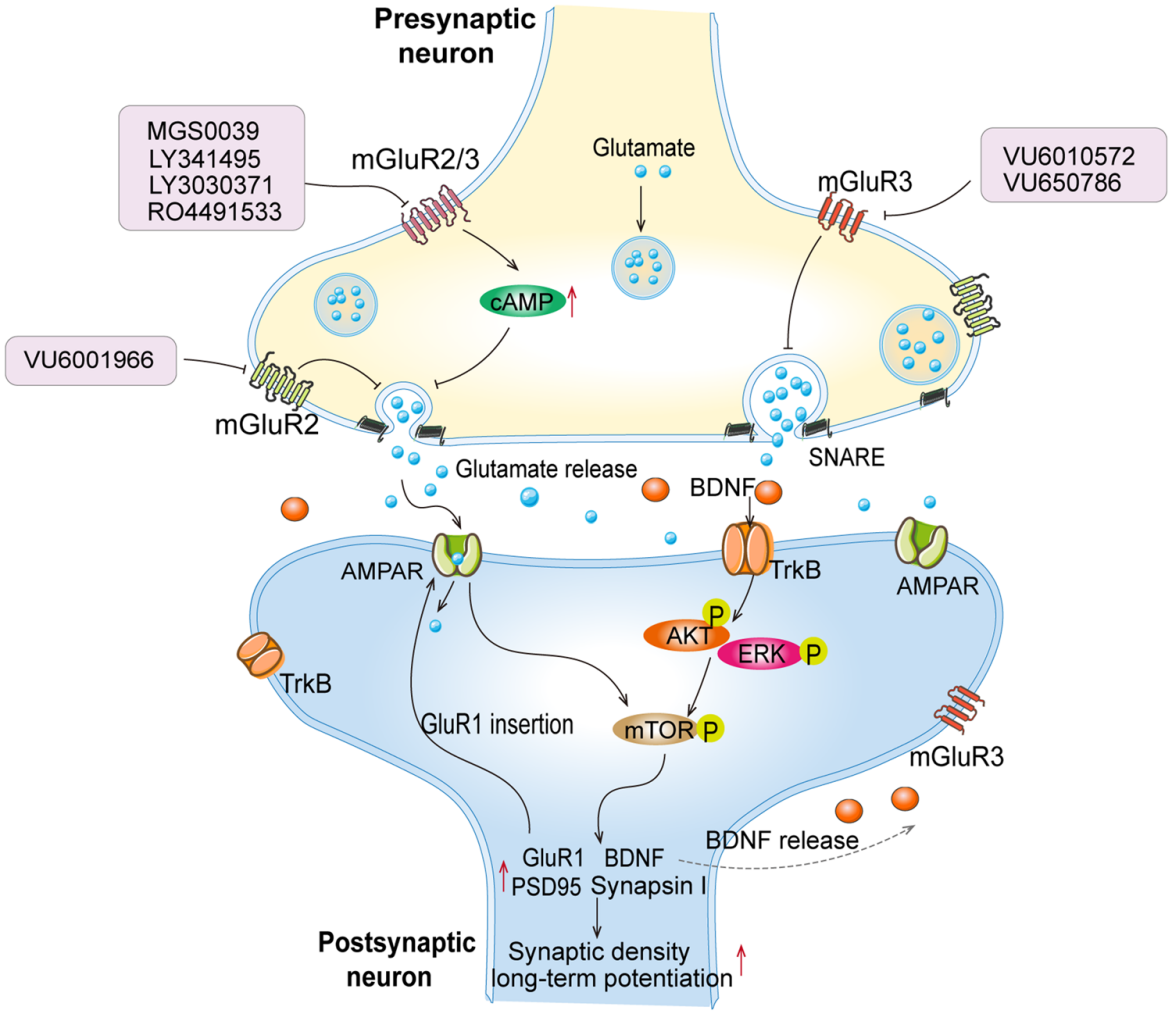
Schematic representation of the mechanism of antidepressant-like action mediated by mGluR2/3 inhibitors. Briefly, the antagonists and NAMs including MGS0039, LY341495 and VU6010572, target at mGluR2 or/and 3 which increases the release of glutamate by inhibiting the activity of cAMP in the presynaptic neuron. While in depression state the extracellular glutamate level is decreased and so does its binding to the AMPAR receptor. The latter further reduces the phosphorylation of mTOR and affects the expression of downstream genes (including GluR1, BDNF, PSD95 and Synapsin I), resulting in a damage of synaptic plasticity and long-term potentiation. *cAMP* cyclic adenosine monophosphate, *SNARE* Soluble N-ethylmaleimide-sensitive factor attachment protein receptor

NAMs antagonize noncompetitively the activity of the orthosteric ligand (Hampson et al. 2008). Therefore, mGluR2/3 NAMs show similar antidepressant-like effects to those of mGluR2/3 antagonists. It was reported that mGluR2/3 NAMs are able to reverse passive coping behavior in FST (Joffe et al. 2020). Furthermore, RO4491533, a mGluR2/3 NAM, shows a strong and fast antidepressant-like effects in acute tests like FST and tail suspension test (TST) (Campo et al. 2011). Selective mGluR3 NAMs, including VU6010572 and VU650786, have been reported to have ketamine-like antidepressant effects in acute depression models like TST (Engers et al. 2017). Notably, a core symptom of depression, the anhedonia induced by corticosterone treatment or chronic stress stimuli can be reversed by a single treatment with mGluR2 or mGluR3 NAM (Chaki 2021; Joffe et al. 2020). These NAMs exert their effects through distinct mechanisms (Machado-Vieira et al. 2017; Tomasetti et al. 2019) (Please refer to Fig. 1), such as activating unique PFC pyramidal cell ensembles, enhancing thalamocortical transmission and reducing long-term depression (Joffe et al. 2020). mGluR3 NAMs are also found to be efficacious in preventing motivational deficits and changes in the amygdalo-cortical plasticity (Joffe et al. 2019), suggesting the potential utility of mGluR3 NAMs for treating psychiatric disorders. Together, these studies demonstrate that developing selective agents to modulate the activity of mGluR2 and mGluR3 may be a promising approach to addressing depressive symptomology.

### Clinical Trials of mGluR2/3 NAMs and Antagonists in Treatment for Depression

Several mGluR2/3 NAMs and antagonists were launched into clinical trials.

For instance, clinical studies are being conducted with RO4995819 (a mGluR2/3 NAM, also known as RG1578 and decoglurant) by Roche (see http://www.clinicaltrials.gov/ct2/show). The Roche RO4995819 has undergone several safety and tolerability Phase I clinical trials (for full list see http://www.rochetrials.com/resultsByProductGet.action?productName=RO4995819), but no published results are currently available. A Phase II 6-week clinical trials are also underway to assess the effects of RO4995819 as an adjunctive treatment in 357 patients with MDD and find no antidepressant responses compared with placebo (see http://www.clinicaltrials.gov/ct2/show/NCT01457677) (Umbricht et al. 2020). Furthermore, a combined usage of sub-effective dosage of LY341495 and ketamine was efficacious for the depression treatment without producing any ketamine-induced side-effects in patients (Agnieszka et al. 2019), suggesting that combination therapy using mGluR2/3 antagonist and ketamine can reduce the effective dosage of ketamine and its side-effects. TS-161, another mGluR2/3 antagonist, has completed phase I (NCT03919409) trials in 70 healthy volunteers to evaluate its safety profile, tolerability and pharmacokinetics. This agent is presently in phase II studies for TRD at the National Institute of Mental Health (Henter et al. 2021). Although both of these drugs appear to be targeted at depression, to date, little human proof-of-concept data are available with mGluR2/3 antagonists and NAMs. However, the clinical efficacies of these compounds could be predicted by investigating similarities in the neural mechanisms between mGluR2/3 antagonists/NAMs and ketamine.

## Discussion

As a mood disorder, depression has a high morbidity and the number of depressed individuals keeps growing with the increase in social competition and life rhythm speed, especially in developing countries (Ren et al. 2020). Despite major advancements in the pathophysiology of depression in recent years, the neural circuits, cellular and molecular mechanisms underlying depression remain poorly understood and the treatment of depression with currently available antidepressants is inadequate either. It highlights the urgent need for further investigation on the pathogenesis of depression and the development of novel antidepressants.

Over the last two decades, several neural systems are proven to be implicated in depression such as the cholinergic system (Drevets et al. 2013) hypothalamo-pituitary-adrenal (HPA) axis (Machado-Vieira et al. 2014), opioid system (Knoll and Carlezon Jr 2010) and melatonergic system (Alexis Geoffroy et al. 2015). In this context, agents acting on these neural systems have also been made and their antidepressant effects are tested in preclinical and/or clinical trials. Although administration of the modulator of cholinergic system, including scopolamine and VU0255035, induces antidepressant effects, it produces unacceptable side-effects like psychosis (Khajavi et al. 2012; Navarria et al. 2015). Similarly,mifepristone and CP-316, two antagonists of HPA axis, show disappointing results in clinical studies (http://www.inpharmatechnologist.com/Regulatory-Safety/Sanofi-pulls-plug-on-four-Ph-III-drugs2009) (Binneman et al. 2008). The opioid system is an undervalued but a promising target in future studies of depression and one modulator of this system, ALKS-5461, shows positive results in phase II trials and is further evaluated in phase III trials as an adjuvant treatment for TRD (http://phx.corporate-ir.net/phoenix.zhtml?c=92211&p=irolcorporateNewsArticle&ID=18258172013). However, to our limited knowledge, just a few agents acting on this system are reported. As to the melatonergic system, an agonist, ramelteon, shows substantial antidepressant efficacy in preclinical and clinical studies (Bertaina-Anglade et al. 2006; Montgomery and Kasper 2007).

In addition to the neural systems mentioned above, mounting evidence has confirmed that dysregulation of glutamatergic system leads to depression (Lee et al. 2022; Olajide et al. 2021). Furthermore, agents acting on the glutamatergic system are efficacious for treating depression. For example, ketamine, the most concerned iGluR(NMDAR) modulator, is highly efficacious for depression including TRD, though it causes unfavorable side-effects. Other iGluR modulators, including GluN2B-specific NMDA receptor antagonists (CP-101/MK-0657) (Ibrahim et al. 2012; Preskorn et al. 2008) and NMDA receptor glycine-site partial agonists (D-cycloserine/GLYX-13) (Depression 2015; Phase), are also under various stages of clinical trials and the results appear to be acceptable to some extent. Under the encouragement of the robust antidepressant effects of ketamine, an increasing number of compounds targeting mGluRs have been tested (Cross et al. 2018; Moridi et al. 2020), and these compounds seem to be the most promising agents under studies for depression among the modulator of glutamatergic system (Dogra and Conn 2021). Notably, compared to other mGluRs, mGluR2/3 is a more specific target for developing novel antidepressants (Dogra and Conn 2021). In fact, the antagonist/NAM of mGluR2/3 shows fast-acting and sustained antidepressant-like effects with no ketamine-like side-effects produced. However, it should be noticed that the safety and efficacy of these compounds (particularly for those that have not yet undergone clinical trials) need to be further verified in preclinical and clinical investigations, as to date, relevant data are not sufficiently comprehensive.

Although several mGluR2/3 antagonists/NAMs show great therapeutic potential for the treatment of depression in preclinical investigations, the outcomes of clinical trials were not particularly encouraging (Umbricht et al. 2020). The possible reasons might be: (I) these drugs have poor gastrointestinal permeability resulting in low oral bioavailability (Holly, LaCrosse, & Hillhouse); (II) both mGluR2/3 antagonists and NAMs selectively target at specific sites (mainly mGluR2/3), but the pathogenesis of depression is multifactorial, thus limiting the antidepressant effects of these agents. At the same time, it highlights the importance of a combined usage of drugs with different mechanisms of action in the treatment of depression; (III) current mGluR2/3 antagonists and NAMs lack specificity in brain regions related to depression.

## Conclusion

Collectively, we remain encouraged by this area of research despite the mixed results and failures. Compounds discussed above selectively acting on mGluR2 and/or mGluR3 have been shown to possess rapid and prolonged antidepressant-like effects with fewer side-effects in preclinical or clinical studies. The advent of these agents has shed valuable light on novel treatment avenues and advanced the ultimate goal of developing much-needed, novel, rapid-acting, safe, and effective treatment options for the millions of individuals worldwide suffering from depression.

## Data Availability

All the data and materials are available.
